# 

**Published:** 1818-07

**Authors:** 


### THE LONDON MEDICAL AND PHYSICAL JOURNAL;

CONTAINING

ORIGINAL CORRESPONDENCE of EMINENT PRACTITIONERS,

AND THE

EARLIEST INFORMATION ON SUBJECTS CONNECTED WITH

*MEDICINE, SURGERY, CHEMISTRY, PHARMACY, BOTANY, AND NATURAL HISTORY.*

*Successively conducted by*

T. BRADLEY, M.D.

A. F. M. WILLICH, M.D.
R. BATTY, M.D.
A. A. NOEHDEN, M.D.
J. ARNEMAN, M.D.
J. P. PFAFF, M.D.
J. ADAMS, M.D.
W. SHEARMAN, M.D.
W. ROYSTON, Esq.

SAMUEL FOTHERGILL, M.D.

AND

WILLIAM HUTCHINSON, Esq.
SURGEON;

ASSISTED BY EMINENT GENTLEMEN, IN THE VARIOUS BRANCHES OF THE PROFESSION, AS WELL IN ENGLAND AS IN FRANCE, GERMANY, ITALY, AND THE UNITED STATES.

VOL. XL.

FROM JULY TO DECEMBER, 1818.

Surgeon Genl's Office LIBRARY. 5746 Washington D.C.

Et quoniam variant morbi, variabimus artes;
Mille mali species, mille salutis erunt.

*LONDON:*

PRINTED FOR THE PROPRIETORS,

BY JAMES ADLARD AND SONS, 23, BARTHOLOMEW-CLOSE;

PUBLISHED BY J. SOUTER, NO. 73, ST. PAUL'S CHURCH-YARD; AND MAY BE HAD OF ALL BOOKSELLERS.

W1
LO31

### BOOKS IN THE PRESS.

In the press, and will speedily be published, a second edition of Dr. W. Philip's Experimental Inquiry into the Laws of the Vital Functions, and the Nature and Treatment of Internal Diseases.

Mr. Carmichael, of Dublin, will shortly publish, Observations on the Symptoms and Specific Distinctions of Venereal Diseases; interspersed with hints for the more effectual prosecution of the present inquiry into the uses and abuses of mercury in their treatment.

### CATALOGUE OF MEDICAL BOOKS.

Elements of Chemical Science, as applied to the Arts and Manufactures and Natural Phenomena. By J. Murray. Second edition, with additions. 12mo. 8s.

A Treatise on the Venereal Disease. By J. Hunter. With an Introduction and Commentary, by J. Adams, M.D. Second edition, with additions, comprehending the late Controversies concerning the Mode of Curing that Disease. 8vo. 14s.

A Guide to Botany; or a Familiar Illustration of the Principles of the Linnæan Classification. With coloured Engravings. By J. Mellar, M.D. 12mo. 7s.

Minutes of Cases of Cancer. Part II. Being Further Reports of Cancerous Cases successfully treated by the New Mode of Pressure; with Observations on the Nature of the Disease, as well as the Method of Practice. By S. Young. 8vo. 9s.

A Practical Guide to the Management of the Teeth; comprising a Discovery of the Origin of Caries or Decay of the Teeth, with its Prevention and Cure. By L. S. Purmly, Dental Professor. 12mo. 5s. 6d.

A Practical Essay on Chemical Re-agents or Tests; illustrated by a Series of Experiments. By F. Accum. 12mo. Second edition. 9s.

### *TO CORRESPONDENTS.*

*Several Communications and Works of Analysis have come to hand, which will be inserted in their due course.*

*Our Correspondents will please to be particular in the Address of their Communications, which should be thus,*—" To the Editors of the London Medical and Physical Journal, at Mr. Souter's, No. 73, St. Paul's Church Yard; or at Mr. Adlard's, 23, Bartholomew-Close."

